# Friends and relatives: insight into conformational regulation from orthologues and evolutionary lineages using KIF and KIN

**DOI:** 10.1039/d4fd00018h

**Published:** 2024-02-29

**Authors:** Dariia Yehorova, Rory M. Crean, Peter M. Kasson, Shina Caroline Lynn Kamerlin

**Affiliations:** a School of Chemistry and Biochemistry, Georgia Institute of Technology USA skamerlin3@gatech.edu; b Department of Chemistry-BMC, Uppsala University Sweden; c Department of Biomedical Engineering, University of Virginia USA; d Department of Cell and Molecular Biology, Uppsala University Sweden; e Departments of Chemistry & Biochemistry and Biomedical Engineering, Georgia Institute of Technology USA pkasson3@gatech.edu

## Abstract

Noncovalent interaction networks provide a powerful means to represent and analyze protein structure. Such networks can represent both static structures and dynamic conformational ensembles. We have recently developed two tools for analyzing such interaction networks and generating hypotheses for protein engineering. Here, we apply these tools to the conformational regulation of substrate specificity in class A β-lactamases, particularly the evolutionary development from generalist to specialist catalytic function and how that can be recapitulated or reversed by protein engineering. These tools, KIF and KIN, generate a set of prioritized residues and interactions as targets for experimental protein engineering.

## Abstract 2

We have developed novel tools to characterize evolutionarily conserved non-covalent interactions in proteins. We showcase their application to understanding substrate specificity in class A β-lactamases, with potential impact for protein engineering.

## Graphical abstract

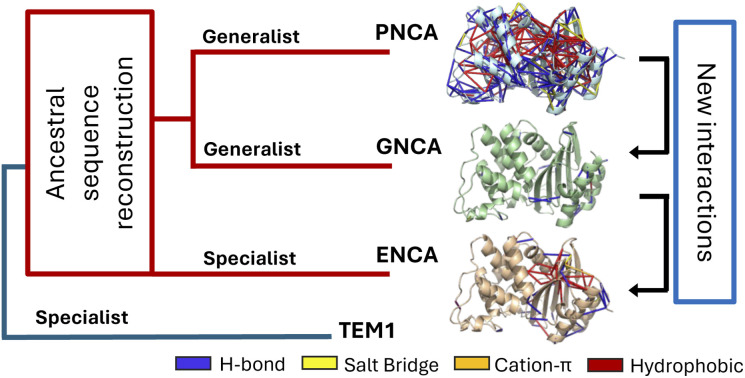

## Introduction

Conformational regulation of protein function is critical to understanding molecular recognition, biological signal transduction, and enzyme substrate specificity, but it has remained challenging to obtain accurate prospective predictions using either computational or experimental approaches. Substantial advances in understanding conformational regulation have been achieved by studying evolutionary families and ancestral lineages.^1–4^ In parallel, improved computational tools to extract insight from conformational ensembles and protein flexibility have yielded an improved understanding of mechanistic regulation.^5–7^ Here, we show how tools that parse evolutionary data on structural templates can be combined with ones that parse conformational data. We illustrate this using tools that we have recently developed and released, Key Interactions Finder (KIF)^8^ for relating noncovalent interactions in structural ensembles to particular outcomes of interest and Key Interaction Networks (KIN)^9^ for analyzing evolutionary groups in the context of protein structures and noncovalent interaction networks. Together, these tools help prioritize residues and networks of interactions for modulating enzyme substrate specificity, in this case generalist *versus* specialist activity of a β-lactamase enzyme.

Extensive sequence-based studies of protein evolutionary groups have yielded both statistical measures of residue–residue coupling and more complex machine-learning analyses of structure and function.^10–16^ Indeed, a major component of sophisticated protein structure prediction algorithms such as AlphaFold^17^ is inference based on multiple sequence alignments. Such approaches have also been used to predict enzyme substrate profiles.^18,19^

Conversely, there is also a rich history of structure-based computational analysis. Molecular dynamics simulations of protein dynamics and conformational ensembles provide one major component of this, but other approaches such as elastic-network models, and rigid-body decompositions have also been used to great effect. In addition to conformational inference, various measures of residue–residue and residue–substrate coupling have been designed and applied. In some cases,^16,20^ these have been combined with evolutionary information as well.

Bacterial class A β-lactamases are clinically important enzymes that mediate drug resistance. These enzymes bind β-lactam substrates including many antibiotics, acylate them to form an acylenzyme intermediate, and then facilitate deacylation and hydrolysis of the β-lactam ring. β-Lactamases also constitute a well-studied test system for conformational regulation, both orthosteric and allosteric, and ancestral sequence reconstruction. Extensive work on class A β-lactamases has suggested that the Ω-loop (residues 164–179 in canonical numbering) plays a particular role in substrate specificity but also that there exist allosteric pathways^21,22^ that can modulate specificity and activity by altering conformational equilibria rather than simply changing static structure.^23,24^ Thus, conformational dynamics and allosteric effects are likely particularly relevant for understanding substrate specificity in this system.

**Fig. 1:**
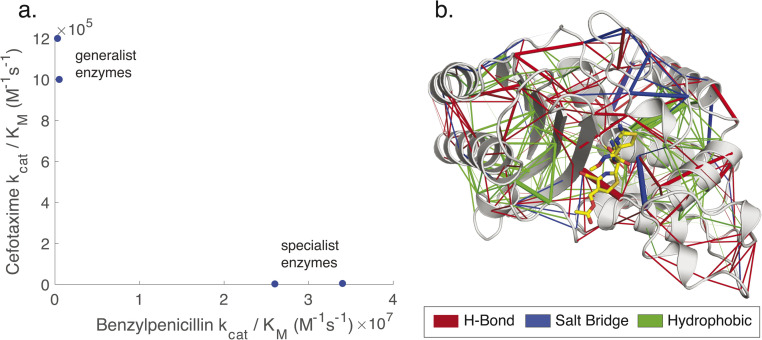
Non-covalent interaction networks as a means to probe generalist *versus* specialist activity in β-lactamases. Panel (a) shows the catalytic efficiencies of two ancestral generalist enzymes (GNCA and PNCA) *versus* one more recent ancestral specialist and one contemporary specialist enzyme (ENCA and TEM-1) on two β-lactam substrates. The names for ancestral enzymes derive from the following abbreviations: ENCA – Enterobacterial Common Ancestor; GNCA – Gram-Negative Common Ancestor; PNCA – (Gram-) Positive and Negative Common Ancestor. Data are replotted from prior work.^25^ Generalist enzymes have similar efficiencies for both substrates, whereas specialist enzymes have higher efficiency and *k*_cat_ for benzylpenicillin and lower for cefotaxime. Panel (b) shows a noncovalent interaction network, rendered as network edges on the TEM-1 structure. The edge weight can be set to reflect either relationship to a target variable (correlation to catalytic permissivity is rendered here) or conservation over an evolutionary group. Interactions can also be separated by type: here hydrogen bonds are rendered in red, salt bridges in blue, and hydrophobic contacts in green.

Due to the availability of such data, we have chosen to demonstrate the combined use of KIF and KIN to probe the evolution of noncovalent interaction networks (Fig. 1) across evolutionary lineages of class A β-lactamases with altered substrate specificity. In particular, we leverage TEM-1 and three reconstructed ancestral β-lactamases, comparing to a curated set of contemporary β-lactamases used in our prior work.^9^ Of the three reconstructed enzymes, one mimics TEM-1 in displaying efficient hydrolysis of benzylpenicillin substrates yet inefficient hydrolysis of cefotaxime (“catalytic specialists”), while two more distant predicted ancestors have approximately similar catalytic efficiencies of benzylpenicillin and cefotaxime substrates (“catalytic generalists”),^25^ as shown in Fig. 1. These reconstructed enzymes have been well characterized biochemically, structurally, spectroscopically, and computationally.^7,25–28^ They share high structural conservation, have similar catalytic activities towards β-lactam antibiotics as the average modern enzyme but broader substrate scope than modern β-lactamases such as TEM-1. Of note, conformational dynamics have been suggested to be important in controlling both substrate scope and also in facilitating design of *de novo* active sites with anthropogenic activities in these β-lactamases. These pairs of enzymes were used to help identify noncovalent interactions that might be associated with conformational regulation of catalytic specialization. At the same time, all three ancestral β-lactamases were compared to contemporary β-lactamases to identify systematic differences between these groups.

## Results and discussion

**Fig. 2:**
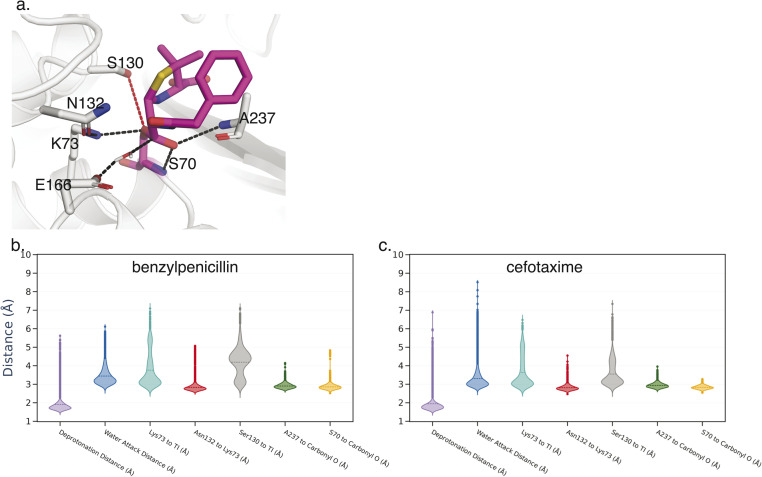
Feature selection for specialist *versus* generalist enzymes. Panel (a) shows seven candidate catalytic distances rendered on the TEM-1 structure, and panels (b) and (c) show violin plots measured on simulation trajectories of TEM-1 in acyl-enzyme intermediate with either benzylpenicillin or cefotaxime. Distances were selected for inclusion if the mode was equal or more favorable with benzylpenicillin, since the TEM-1 catalytic efficiency for benzylpenicillin is ∼1000-fold greater.

Key Interactions Finder (KIF) identifies noncovalent interactions by mutual information, linear, or rank correlation with a target variable. To select such a variable, we performed molecular dynamics simulations (see Methodology section) of the acyl-enzyme intermediate for β-lactam hydrolysis by TEM-1 and three selected ancestral β-lactamases as discussed in the Introduction. The resulting trajectories were analyzed for conformational differences informative of substrate specificity as detailed below. The acyl-enzyme intermediate was chosen to study substrate specificity, because the deacylation reaction is often rate-limiting and tends to vary much more with substrate than the acylation reaction for these enzymes.^29–31^ In fact, most clinical inhibitors of β-lactamases readily undergo acylation but not deacylation. We considered 7 active-site distances previously associated with catalysis of the deacylation reaction (Fig. 2). We used as our inclusion criterion that each distance should be more catalytically favorable for deacylation in terms of positioning the water for nucleophilic attack and stabilization of the deacylation transition state in the TEM-1:benzylpenicillin acyl complex than in the TEM-1:cefotaxime acyl complex since the former deacylation rate is substantially faster. 6 of the 7 distances met this criterion, and their unweighted sum was used as the target variable for identifying relevant noncovalent interactions.

**Fig. 3:**
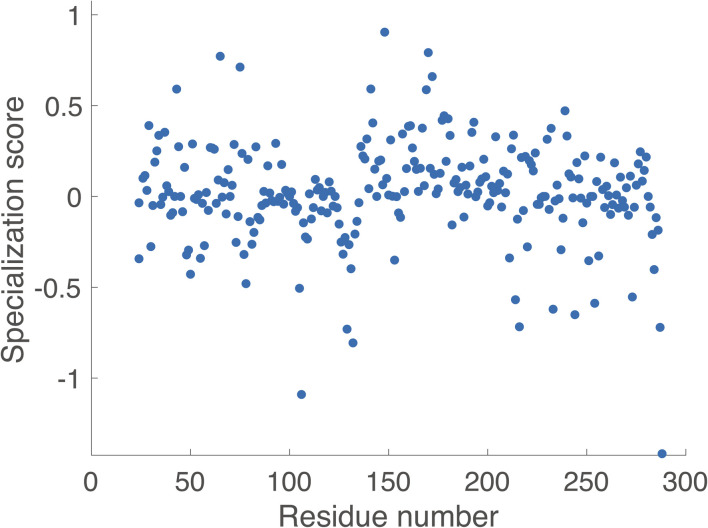
Predicted specialization scores. Specialization scores are plotted as a function of residue number. These reflect the aggregate interactions for a given residue that correlate with specialization for benzylpenicillin (large positive numbers), no strong preference (low absolute values), or generalization towards ancestral activity (large negative numbers).

**Fig. 4:**
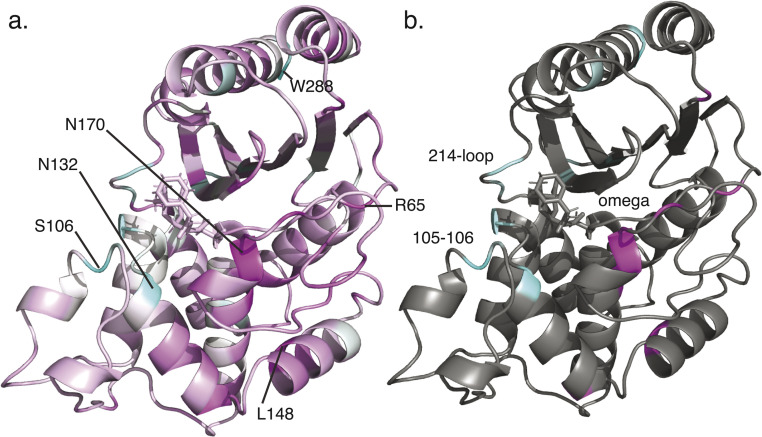
Structural rendering of predicted influential residues. Panel (a) shows a cyan–white–magenta coloring by specialization score, with residues predicted influential in ancestral generalist interactions in cyan and residues predicted influential in contemporary specialist interactions in magenta, with the top 5 residues and one likely artifact at the C-terminus labeled. Panel (b) shows only the residues with absolute value specialization score >0.5.

Using KIF, we first identified noncovalent interactions mostly predictive of favorable catalytic distances and then examined conformational regulation of catalysis. In order to probe specificity differences between benzylpenicillin and cefotaxime substrates, we used the metric *∂* = *C*_benzylpenicillin_ – *C*_cefotaxime_, where *C*_i_ denote correlation scores from KIF corresponding to interaction matrices. We then determined a composite score per residue (see Methodology section) and defined the specialization score *S* = 〈*∂*_specialist_〉 − 〈*∂*_generalist_〉, where the *∂* values per residue were further averaged over the “catalytic specialist” enzymes that have 1000-fold greater *k*_cat_/*K*_M_ for benzylpenicillin than for cefotaxime and the “catalytic generalist” enzymes that have approximately equal *k*_cat_/*K*_M_ for the two substrates. These specialization scores are plotted in Fig. 3 and rendered on the TEM-1 structure in Fig. 4. These residues are therefore predicted to participate in interactions that determine catalytic specificity *versus* generality for TEM-1 and its ancestors.

It is noteworthy that the top-scoring residues emerging from this analysis include positions on the Ω-loop and the 214–220 loop, both of which have been shown to impact substrate specificity.^32–34^ In addition, the high-scoring 105–106 sites have been identified in clinical variants and prior computational analyses of allosteric pathways.^21,35^ Additional top-scoring residues such as Asn132 (strongly conserved but subtle changes in position are implicated in substrate specificity changes^36^), Leu148 (which we predict to be important in attaining specialist function), and Arg65 (which we predict to be important in attaining specialist function) are less well documented and may represent novel predictions.

**Fig. 5:**
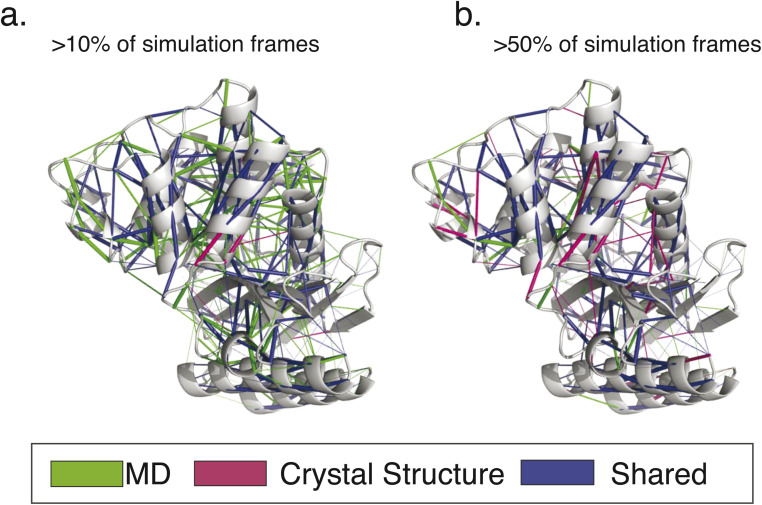
Conserved interaction networks for modern class A β-lactamases. The conserved interaction networks for modern class A β-lactamases are plotted using (a) interactions present in >10% of simulation frames for each simulated enzyme and (b) interactions present in >50% of simulation frames. Interactions are rendered as edges projected onto the TEM-1 structure. As can be appreciated, more static interactions show a greater overlap with those calculated based on crystallographic structures only, while more transient interactions have the greatest sensitivity of detection. Renderings reproduced from our prior work.^9^

**Fig. 6:**
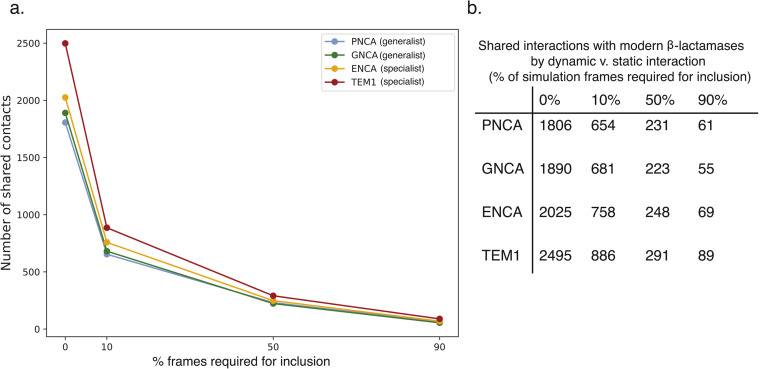
Shared interactions between specialist or generalist enzymes and a conserved network for modern β-lactamases. The number of interactions is plotted in (a) and tabulated in (b) as a fraction of a cutoff for dynamism: the fraction of simulation frames in which an interaction must be present to be included. Shared interactions are much more prominent when transient interactions are included, reinforcing the importance of dynamics in ancestral enzyme function.

For additional insight into the development of catalytic specialization and the mechanism of ancestral catalytic generality, we leveraged the Key Interaction Networks (KIN) tool as follows. A network of conserved noncovalent interactions was calculated for modern β-lactamases and projected onto the TEM-1 structure (Fig. 5). Similarly, conserved noncovalent interactions were calculated for all three ancestral enzymes and for just the two ancestral catalytic generalists and projected onto TEM-1 as a common reference. Interaction networks were calculated with a sliding cutoff using either just “static” interactions preserved from crystallographic structures or “dynamic” interactions that were present in a fraction of molecular dynamics simulation frames (Fig. 6). Many of these relate to stabilization of core structural motifs and likely help position the active site. Interestingly, the overall number of shared interactions does not show an obvious trend among specialist and generalist ancestors, likely reflecting this structural requirement. However, the number of shared interactions consistently increased as more dynamic interactions were allowed in the network. This qualitatively agrees with the emerging understanding of ancestral enzymes – that dynamics and conformational flexibility are key to permitting catalysis of a broader range of substrates.^37–40^

**Fig. 7:**
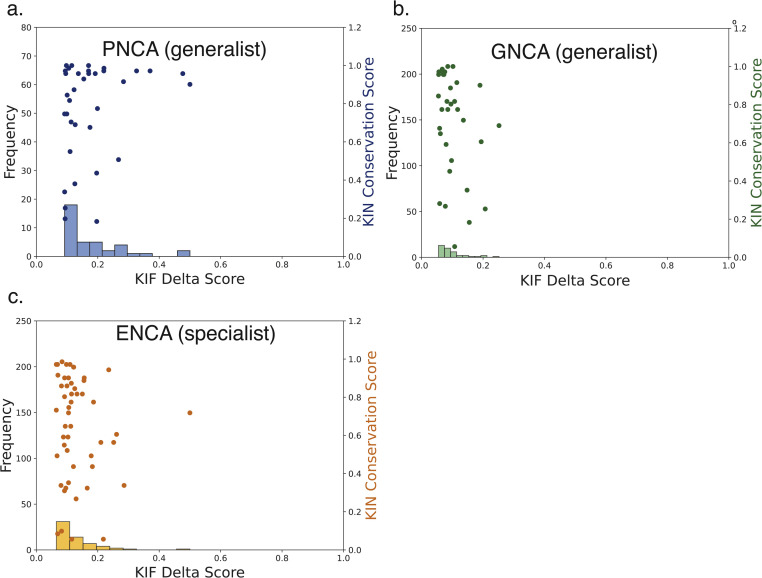
Most shared interactions have low specialization scores. Scatter plots of conservation *versus* delta scores and histograms are plotted at a 50% cutoff for dynamism. Most shared interactions between each ancestor and modern β-lactamases are not implicated in ligand specificity, reinforcing the idea that these stabilize overall structure and catalytic competence.

**Fig. 8:**
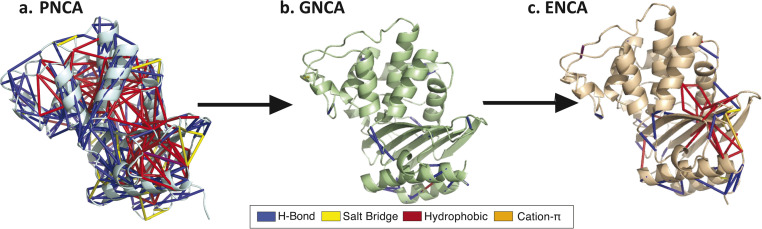
Interaction subnetworks present in generalist ancestors may guide to reconstruction. The shared interaction network present in the generalist PNCA is plotted in (a) and then new interactions along the evolutionary lineage are plotted for (b) GNCA and (c) ENCA. We speculate that these new subnetworks may impact conformational regulation important for specialist *vs.* generalist enzyme function. In these renderings, blue edges denote hydrogen bonds, yellow edges salt bridges, red edges hydrophobic interactions, and orange edges cation–π interactions.

As would be expected, the key shared interaction networks generally have low KIF specialization scores *S* (Fig. 7); instead, residues that score highly for specialization in fact have different interactions between the ancestral and modern β-lactamases. One would expect that residues and interactions with high specialization scores for differentiation of generalist enzymes into specialists would be in the conserved network of modern β-lactamases but not be found among the shared ancestral and modern interactions. Thus, this application of KIF identification yields targets for change in specialization, whereas this application of KIN yields important conserved interactions that should not be changed. However, KIN can be used to identify networks of interactions in specialist enzymes that are missing from generalists. These, rendered in Fig. 8, may represent opportunities for targeted reconstruction of ancestral function or targeted specialization of ancestral enzymes.

The identification of particular interactions that correlate with specialist *versus* generalist enzyme function provides an obvious target for protein engineering efforts to modulate substrate spectrum. We speculate that such interactions could be engineered to restore ancestral generalist function to a specialist enzyme or *vice versa*. Clearly, the hit rate of such attempts would be far from perfect, but KIF and KIN provide a means to winnow the vast combinatorial space of available mutations and generate a prioritized list for further protein engineering efforts.

## Conclusions

Together, KIF and KIN comprise a set of tools to identify noncovalent interactions associated with a particular target variable – in this case catalytically permissive distances in molecular dynamics simulations – and to analyze conserved interaction networks across evolutionary groups. These can be combined to identify interactions of particular relevance to a process such as alignment of active-site catalytic residues and follow those interactions across evolutionary groups, relating them to conserved evolutionary networks. The resulting predictions of influential residues and contacts can help prioritize mutations to engineer substrate generality onto specialist enzymes or conversely engineer substrate specificity onto ancestral generalist enzymes.

## Methodology

### Structure selection

This work considered two sets of initial structures: 3 β-lactamases obtained *via* ancestral sequence reconstruction (ASR) and a set of 69 modern class A β-lactamase structures. The ASR structures include ENCA (3ZDJ), GNCA (4B88), and PNCA (4C6Y), and were selected based on evolutionary relations between the selected species illustrated in previous literature.^25^ The set of modern β-lactamases consists of the following structures: 1BSG, 1BUE, 1DY6, 1E25, 1G6A, 1GHP, 1HTZ, 1HZO, 1M40, 1N9B, 1YLW, 2CC1, 2P74, 2QPN, 2WK0,2ZD8, 2ZQ7, 3BFF, 3BYD, 3LEZ, 3P09, 3P98, 3QHY, 3TSG, 3V3R, 3V3S, 3W4P, 3W4Q, 3ZNW, 3ZNY, 4EUZ, 4EWF, 4MXG, 4QU3, 4UA6, 4YFM, 5A92, 5E2E, 5E43, 5F82, 5GHX, 5HW3, 5NE2, 5NJ2, 5NPO, 5TFQ, 5VPQ, 6AFM, 6BN3, 6BU3, 6J25, 6MK6, 6MU9, 6NIQ, 6NJ1, 6PQ9, 6QWA, 6QWB, 6TD0, 6W2Z, 6W34, 6WGP, 6WGR, 6WIP, 6WJM, 7A6Z, 7BDR, 7DDM, 7QLP.

### Parametrization

Force field parameters for the tetrahedral intermediate forms of benzylpenicillin and cefotaxime were generated to be compatible with Amber and our choice of protein force field (Amber ff14SB).^41^ To generate each parameter a model of both tetrahedral intermediates was generated using the PDB structures 1IYQ and 1IYO,^42^ which contain a structure of benzylpenicillin and cefotaxime respectively in the tetrahedral intermediate form, bound to the Toho-1 β-lactamase. The backbone of the serine amino acid that is covalently linked to the drug molecule was capped using acetyl (ACE) and *N*-methylamide (NME) capping groups. Partial charges were calculated using the restrained electrostatic potential (RESP) protocol using the R.E.D. Server.^43^ All other parameters for the backbone and Cβ atom were taken directly from the ff14SB force field,^41^ whilst the remainder of the molecule was described using GAFF2.^44^ The parameters used are provided in an accompanying GitHub repository: https://github.com/kamerlinlab/friends_and_neighbors.

### Structure preparation and simulation details

Preparation of the modern β-lactamase set along with the computational details on the corresponding simulation details has been described in our previous publication.^9^ The structures and data are also made available on GitHub at https://github.com/kamerlinlab/KIN.

To build the structures of the ancestral enzymes and TEM-1 containing a tetrahedral intermediate form from the substrate benzylpenicillin and cefotaxime, the structures 1IYQ and 1IYO^42^ were used as templates. These two structures have benzylpenicillin and cefotaxime respectively bound to the active site of TOHO-1 β-lactamase. These structures were therefore aligned to each ancestral structure and TEM-1 in order to get the coordinates for the tetrahedral intermediate alongside the catalytic water.

Ancestral sequences were prepared for molecular dynamics (MD) simulations using the same preparation protocol as described in previous work.^9^ To be consistent with our prior work, conventional MD simulations of the unliganded ancestral enzymes were performed for 5 × 100 ns each. For the MD simulations of each ancestral enzyme and TEM-1 with either of the two tetrahedral intermediates, 10 replicas of 200 ns each were performed for each structure. All MD simulations were performed using the Amber simulation package, with the Amber ff14SB force field^41^ and TIP3P water model. Simulations were run using a 2 fs timestep in an *NPT* ensemble (300 K and 1 atm) and followed the same equilibration and production protocol/settings as in our prior study.^9^

### KIF analysis

Key Interactions Finder (KIF)^8^ is a Python program to analyze MD simulations and identify the most strongly associated non-covalent interactions to a given descriptor/target variable. In this case the descriptor was used to define the degree to which a given conformation was catalytically competent. We summed together 6 heavy atom distance measurements whereby a lower value of each distance would mean a more catalytically competent position. These 6 distances are: (1) the catalytic water to the E166 side chain oxygen distance; (2) the catalytic water to the carbonyl group carbon on the tetrahedral intermediate; (3) the K73 side chain nitrogen hydrogen bond to the carbonyl group; (4) the hydrogen bond distance between the N132 side chain and K73 side chain; (5) the A237 hydrogen bond to the carbonyl group; and (6) the S70 main chain hydrogen bond to the carbonyl group. As shown in Fig. 2, the S130–carbonyl group distance was also measured but failed our inclusion criterion of reproducing the benzylpenicillin *vs.* cefotaxime substrate trend for TEM-1.

For the KIF analysis, we used 10 000 frames per system (frames taken every 0.2 ns), with non-covalent interactions of type hydrogen bond, salt bridge and hydrophobic used, and any interaction with an occupancy <25% was excluded from the analysis. The statistical analysis module was used to calculate the per interaction scores. As the target variable is continuous, the regression module was used with the linear correlation used to generate the per interaction scores.

### KIN analysis

Key Interaction Networks^9^ is a tool that can analyze either experimentally-derived PDB structures or molecular dynamics trajectories for evolutionary groups of proteins, yielding conserved networks of static or dynamic interactions. This software is available on GitHub at https://github.com/kamerlinlab/KIN. Here, we used interaction information obtained from unliganded simulations for all considered structures; the underlying hypothesis is that the dynamics in the unliganded state correlate strongly with the overall dynamics in the acylenzyme state. Ancestral sequences and modern proteins were aligned to TEM-1 (PDB 1M40)^45^ using the multiple sequence alignment tools in Modeller.^46^ TEM-1 was chosen as a representative structure for a comparison between two sets of structures due to its evolutionary relationship to the chosen ancestral structures and chemical relevance as a catalytic specialist.

All modern β-lactamases were aligned to TEM-1 and were used to produce a conservation network using KIN. The conservation network provides a relative conservation score per interacting residue pair based on the abundance of each interaction among the structures. It also provides additional information on the interaction of interest, such as the location of the participating residues and whether it belongs to one of the following 6 types: H-bonding, hydrophobic, salt bridge, π–π, cation–π or van der Waals. Conservation networks were generated based on residue interaction networks (RIN) constructed from MD data with a varying dynamic cutoff. This cutoff determines how persistent interaction must be among the MD trajectories to be considered for further analysis. For example, a cutoff of 50% would imply that the interaction was present in at least 50% of the simulation frames. Conservation networks with cutoffs of 0%, 10%, 50%, and 90% were considered to represent interactions that carry dynamic and static character.

All ancestral structures were individually aligned to TEM-1 to obtain an RIN that represents the ancestral protein interactions projected onto the TEM-1 structure for further comparison. Each of the RINs was constructed from MD simulations with a dynamic cutoff of 0%, 10%, 50% and 90%. Independent ancestral RINs were compared among themselves as well as to the conservation network of the modern β-lactamases. Additionally, the evolutionary-driven contribution of interactions was investigated by filtering out unique interactions that appear in ancestral sequence reconstruction along the evolutionary tree. Lastly, KIN scores were compared to the *∂* values obtained from KIF analysis.

## Conflicts of interest

There are no conflicts to declare.
